# Influence of low-cost Thai leucoxene minerals on the growth, bioactive compounds, and antibacterial activities of *Chrysanthemum indium* L. cuttings in in vitro culture

**DOI:** 10.1038/s41598-024-60131-5

**Published:** 2024-04-25

**Authors:** Sorapong Pavasupree, Nattapong Chanchula, Narittaya Nunya, Sirinya Kashima, Pariya Na Nakorn, Esther Thongaram, Yayoi Shindo, Atipong Bootchanont, Chakkaphan Wattanawikkam, Russameeruk Noonuruk, Kamonporn Srilopan, Porramain Porjai

**Affiliations:** 1 Department of Materials and Metallurgical Engineering, Faculty of Engineering, Rajamangala University of Technology Thanyaburi, Pathum Thani, Thailand; 2 Expert Center of Innovative Agriculture (InnoAg), Thailand Institute of Scientific and Technological Research (TISTR), Technopolis, Khlong Ha, Khlong Luang, Pathum Thani, Thailand; 3 Department of Biotechnology, Faculty of Science and Technology, Thammasat University KlongNueng, Klong Luang, Pathum Thani, 12120 Thailand; 4 Division of Physics, Faculty of Science and Technology, Rajamangala University of Technology Thanyaburi, Pathum Thani, Thailand; 5 Smart Materials Research Unit, Rajamagala University of Technology Thanyaburi, Pathum Thani, Thailand

**Keywords:** Chlorophylls, Antioxidant enzyme activities, Phenolic compounds, Antibacterial abilities, Leucoxene mineral, Biotechnology, Plant sciences, Materials science

## Abstract

The effects of low-cost Thai leucoxene mineral (LM) at different concentrations (10, 20, 30, 40, 50, and 60 mg/L) on the growth and antibacterial properties of *Chrysanthemum indium* L. cuttings under in vitro were evaluated. The primary chemical composition of LM was approximately 86% titanium dioxide (TiO_2_), as determined by dispersive X-ray spectroscopy. The crystalline structure, shape, and size were investigated by X-ray diffraction and scanning electron microscopy. LM at 40 and 50 mg/L significantly increased plant height, leaf number, node number, and fresh and dry weight. These growth-promoting properties were accompanied by improved chlorophyll and carotenoid contents and antioxidant enzyme activities and reduced malondialdehyde levels. Additionally, LM treatment at 40 and 50 mg/L had positive effects on antibacterial activity, as indicated by the lowest minimum inhibitory concentration (MIC) and minimum bactericidal concentration (MBC) values. The high levels of phenolic compounds in the plants contributed to the MIC and MBC values. In conclusion, these findings provide evidence for the effectiveness of LM in enhancing the growth of *Chrysanthemum* plants in in vitro culture and improving their antibacterial abilities.

## Introduction

*Chrysanthemum indium* L. (*C. indium*) is in the *Asteraceae* family and is an aromatic and medicinal plant generally found in Thailand, Korea, Japan, and China^1,2^. *C. indium* is also a highly valued ornamental crop worldwide ^3^. This flower holds cultural significance, with billions of floral stems sold annually. *C. indium* is cultivated in various colors, sizes, and forms of composite *Chrysanthemum* flowers by incorporating different combinations, concentrations, and types of anthocyanins, carotenoids, and chlorophyll^2^. In addition, dried *C. indium* (especially the flowers and buds) is regularly applied to treat eye diseases and is used for the preparation of traditional tea^1^. Furthermore, *C. indium* extracts have been used to treat various conditions, such as hypertension and respiratory and inflammatory diseases^4^. This plant is also known for its various health benefits, including antimicrobial, antibacterial, and anticancer effects^5^.

The propagation of *C. indium* using seeds is challenging due to its long dormancy period and low germination rate^6^. Various factors, including the type of explant, plant size, plant growth regulators, and temperature, can contribute to its slow development or failure to germinate, affecting regularity. Modern biological methods such as micropropagation and mutation breeding via in vitro conditions have been developed to produce higher yields of *C. indium*. Generally, the cultivation of *C. indium* is performed by using shoot cuttings and root suckers^2^. Shoot cutting is a popular and simple method of in vitro propagation because it has high natural mutation rates and is self-incompatible^7–9^. This method also maintains the characteristics and qualities of the mother plant^10^. Nevertheless, this strategy has limitations, including a diminished reproduction rate, a substandard seedling quality, an extended reproduction time, seasonal limitations, limited genetic diversity, and the inability to circumvent cross-incompatibility. Moreover, cuttings frequently sourced from parent plants may succumb to viral infections and degradation, consequently increasing production costs^2^. Thus, enhancing the yield of *C. indium* in tissue culture using new techniques has become a challenging problem for scientists.

Several studies have investigated the use of tissue culture for the large-scale propagation of *Chrysanthemum morifolium* (*C. morifolium*) by exploring novel regeneration pathways^11,12^. Establishing effective strategies to prevent microbial contamination in culture media is crucial. Instead of relying solely on physical sterilization methods such as autoclaving, chemical agents, nanoparticles, or plant extracts, either alone or in combination with autoclaving, can be considered^13^. Furthermore, explants cultivated in vitro serve as exceptionally suitable materials for irradiation purposes. By utilizing in vitro cultures within a confined, disease- and pest-free environment, a substantial quantity of irradiated explants can be accommodated in a limited space. This approach ensures more efficient regeneration than in vivo conditions, enhances the likelihood of obtaining mutated plants, and facilitates a significant acceleration of all stages within the breeding program. In a previous study in which *C. morifolium* was cultured in vitro at a low concentration of 6-benzylaminopurine effectively enhanced the growth of roots and shoots^14^. Interactions between cytokines and auxins are also considered to induce the roots and shoots of *C. indium* plants^15^.

Titanium dioxide (TiO_2_) and TiO_2_-based materials have been widely used in a variety of applications, such as semiconductor materials, water treatment materials^16^, antimicrobial activity^17^, and agriculture^18^. TiO_2_ nanoparticles (TiO_2_ NPs) are applied to plants to enhance their growth and performance. TiO_2_ NPs affect plant morphology, chlorophyll formation, Rubisco enzyme activity, and photosynthesis, improving plant growth. The major benefit of TiO_2_ NPs is their ability to produce reactive oxygen species (ROS)^19^, which play an important role in plant development. TiO_2_ NPs induce chloroplasts in photosynthetic organisms, and since chloroplasts are one of the main establishments that produce ROS in plants^19^, changes in ROS induced by TiO_2_ NPs might be related to changes in chloroplast function. Various studies have reported that TiO_2_ NPs can affect plants. For example, Gohari et al.^20^ reported improvements in the growth, chlorophyll content, carotenoid content, and antioxidant enzyme activity of *Dracocephalum moldavica* (*D*. *moldavica*) with increasing TiO_2_ NP concentrations. Mustafa et al.^21^ reported that TiO_2_ NPs improved bioactive compounds in wheat plants. The combination of TiO_2_ NPs and light intensity could enhance the biomass and leaf thickness of radish, as reported by Vatankhah et al^22^. In recent years, TiO_2_ nanoparticles have garnered attention in *C. indium* cuttings under in vitro culture because TiO_2_ increases the growth efficiency of plants^23^. In addition, TiO_2_ NPs can promote the growth of many plants, such as *Mentha piperita* L. ^24^, *Vicia faba* ^25^, greenhouse-grown cut roses^26^ and *Sesamum indicum* L.^27^. Although TiO_2_ NPs positively affect plant growth, they are expensive due to their difficult synthesis process.

Leucoxene is a versatile mineral that mainly comprises TiO_2_ and impurities such as iron oxide (Fe_2_O_3_), silicon dioxide (SiO_2_), aluminum oxide (Al_2_O_3_), and zirconium dioxide (ZrO_2_). Because of its high TiO_2_ content, leucoxene has been used as a starting material to synthesize TiO_2_ NPs for several applications, such as protective photocatalysts, UV absorbers, and pigments in paints and plastics^28^. The synthesis of TiO_2_ NPs is complicated, and various techniques, such as hydrothermal technology, electrodeposition, and electrospinning, are used^29^. These processes are expensive and time-consuming.

As outlined in the literature, TiO_2_ NPs have been widely used in agricultural applications; however, the direct impact of leucoxene mineral (LM) on plant germination has never been investigated. Here, the objective of this study was to use a low-cost natural LM (< 0.5 USD/kg) instead of TiO_2_ NPs to study its impact on the in vitro growth, antioxidant capacity, and antibacterial properties of *C. indium*. Scanning electron microscopy was used to determine the shape and size of LM, and X-ray diffraction and dispersive X-ray spectroscopy were used to determine its crystalline structure and chemical composition. The effects of different LM concentrations (10, 20, 30, 40, 50, and 60 mg/L) on the morphophysiological and biochemical characteristics of *C. indium* were evaluated and compared to those of a control group. Furthermore, the phenolic compound content, flavonoid content, DPPH radical scavenging capacity, and antibacterial activity of untreated and LM-treated *C. indium* were observed.

## Materials and methods

### Preparation of LM

Sakorn Minerals Co., Ltd. supplied a low-cost natural LM from Prachuap Khiri Khan Province (12.1712° N, 99.8125° E), Thailand. To prepare a powder, the natural LM was ball milled at 250 rpm for 12 min. X-ray diffraction (XRD, PANalytical X'Pert PRO MRD) and dispersive X-ray spectrometry (EDS, model INCA-350) were used to analyze the crystalline structure and chemical composition of the mineral powder. Scanning electron microscopy (SEM; model JSM-5410LV) was used to determine the shape and size of the LM particles^28^.

### Chemical reagents

Potassium phosphate, polyvinylpyrrolidone (PVP), hydrogen peroxide, 2,2-diphenyl-1-picrylhydrazyl (DPPH), sodium phosphate, methionine, riboflavin, ethylenediaminetetraacetic acid (EDTA), nitroblue tetrazolium (NBT), ethanol, Folin–Ciocalteu reagent, gallic acid, malondialdehyde kits, dimethyl sulfoxide, and sodium carbonate were purchased from Sigma Aldrich (St. Louis, MO, USA).

### Plant materials

The plant materials were cultured in a field at the Thailand Institute of Scientific and Technological Research (TISTR). Samples were collected from naturally growing *C. indicum* plants between November and December 2021^23^.

### Stem cutting and cultivation of *C. indicum*

*C. indicum* cuttings were cut to an average length of 1 cm and a diameter of 0.2 cm. These cuttings were subsequently grown in Murashige and Skoog^30^ media supplemented with different concentrations of LM (10–60 mg/L), followed by sucrose (30 mg/L) and Gelrite (3 mg/L). A control group with no TiO_2_ NPs was used. The final pH of the medium was adjusted to 5.7. *C. indicum* was cultured under a light–dark cycle of 16/8 h at 25 ± 2 °C for 8 weeks. The light intensity was set at 60 ± 5 µmol/m^2^/s using fluorescent lights. The characteristics of *C. indicum* were measured every 2 weeks.

## Experimental design

One treatment was conducted on ten cuttings, with six replicates per treatment. Plant height and root, leaf, and node numbers were recorded every two weeks. The fresh shoot weight, root weight, and root length were recorded on the eighth week of cultivation. To determine the dry shoot and root weights, the shoots and roots were dried in a hot air oven at 90 °C for 24 h^23^.

### Total chlorophyll and carotenoid content measurements

Total chlorophyll, chlorophyll *a (Chl* a), chlorophyll b (*Chl* b), and total carotenoid contents were determined using a previously described method^31^. A 50-mg dry leaf sample was mixed with 5 mL of dimethyl sulfoxide (DMSO). The mixture was stored in the dark at 60 °C until the tissue became colorless. The absorbance of the mixture was determined at 480, 647, and 664 nm using a spectrophotometer, with DMSO as the blank. The pigment content was calculated using the following equations:

$$\begin{gathered} Chl{\text{a }}( {{\text{mg}}/{\text{g DW}}}) \, = \, ( {{12}A_{{{664}}} {-}{ 3}.{11}A_{{{647}}} } ) \hfill \\ Chl{\text{b }}( {{\text{mg}}/{\text{g DW}}} ) \, = \, ( {{2}0.{78}A_{{{647}}} {-}{ 4}.{88}A_{{{664}}} }) \hfill \\ {\text{Total chlorophyll }}( {{\text{mg}}/{\text{g DW}}} ) \, = Chl{\text{a }} + Chl{\text{b}} \hfill \\ {\text{Carotenoids }}( {{\text{mg}}/{\text{g DW}}} ) \, = \,( {{1}000A_{{{48}0}} {-}{ 1}.{12}Chl{\text{a }}{-}{ 34}.0{7}Chl{\text{b}}} )/{245} \hfill \\ \end{gathered}$$

where DW is the dry weight.

### Antioxidant enzyme activity measurement

#### Extraction

One gram of plant leaves was homogenized in potassium phosphate buffer (pH 6.8, 10 mM) containing 1% polyvinylpyrrolidone (PVP) using a magnetic stirrer for 10 min. After centrifugation at 6,000 rpm for 20 min, the supernatant was retained to determine ascorbate peroxidase (APX)^32^, catalase (CAT)^32^, and superoxide dismutase (SOD) activities^32^.

#### Ascorbate peroxidase (APX) activity

The activity of APX was evaluated following a previously described method^32^. The enzyme extract was added to an assay mixture consisting of 250 μL of 1 mM ascorbate, 250 μL of potassium phosphate buffer, 250 μL of 0.4 mM EDTA, 190 μL of distilled water, and 250 μL of H_2_O_2_ (10 mM). The absorbance of the sample was determined at 290 nm using a spectrophotometer. An extinction coefficient of 2.8 cm^−1^ mmol^−1^ was used to calculate the results.

#### Catalase activity

CAT activity was determined using a calorimetric method^32^. Three micrograms of crude extract was mixed with 20 mM sodium phosphate buffer (pH 7.5) and 6 mM H_2_O_2_. The final volume of the mixture was one milliliter. The activity of CAT was measured by the decreasing rate of H_2_O_2_ at 240 nm for 3 min using a spectrophotometer. The extinction coefficient was 40 M^–1^ cm^–1^. The results are expressed as mM per min per mg of protein (mM min^-1^ mg^-1^ of protein).

#### Superoxide dismutase (SOD) activity

SOD activity was evaluated by measuring the inhibition of the photochemical reduction of nitroblue tetrazolium (NBT)^32^. Specifically, 50 mL of crude extract was added to the reaction mixture, which was prepared with 130 mM methionine, 0.1 mM ethylenediaminetetraacetic acid (EDTA), 50 mM phosphate buffer (pH 7.8), 0.02 mM riboflavin, and 0.75 mM NBT. The final volume was 3 mL. The mixture was placed under fluorescent lamps (5,000 lx) for 15 min. Then, the absorbance of the mixture was determined at 560 nm using a spectrophotometer. The value of the enzyme required to inhibit NBT photoreduction by 50% was considered one unit of SOD.

### Malondialdehyde level measurement

Lipid peroxidation was monitored by measuring malondialdehyde (MDA) levels according to a previous study^33^. For extraction, 5 mL of 100 g/L trichloroacetic acid was added to fresh tissue (1 g). After centrifugation at 9000 rpm for 20 min at 4 °C, 3 mL of the supernatant was mixed with 3 mL of 0.67% thiobarbituric acid (TBA). The mixture was incubated at 100 °C for 20 min and then placed on ice to cool to room temperature. The absorbance of the samples at 450, 532, and 600 nm was measured with a spectrophotometer to determine the MDA concentration. The results are expressed as nmol/L fresh weight (nmol/L FW).

### Total phenolic content (TPC) measurement

The TPC was estimated using the Folin–Ciocalteu colorimetric method^33^. The reference standard was gallic acid. The sample extract was diluted with 80% ethanol at a ratio of 1:5. Distilled water (0.5 mL) was mixed with 125 μL of Folin–Ciocalteu reagent (10% w/v), followed by 125 μL of the sample. The mixture was left at room temperature for 6 min, after which 1.25 mL of sodium carbonate solution (7%) was added. The absorbance of the sample was evaluated at 760 nm. The results are expressed as mg of gallic acid equivalents per gram of extract (mg GAE/g extract).

### Total flavonoid content (TFC) measurement

The TFC was measured by the colorimeter method^33^. A total of 0.5 mL of plant extract was added to 1.5 mL of methanol, potassium acetate, 1 M 10% aluminum chloride, and 2.8 mL of distilled water. After vortexing and incubation in darkness for 30 min, the absorbance of the solution was evaluated at 415 nm using a microplate reader (SpectraMax, Model i3x Multi-Mode). The reference standard was quercetin; thus, the results are expressed as milligrams of quercetin equivalent per gram of extract (mg QUE/g extract).

### Antioxidant activity measurement

DPPH radical scavenging activity was evaluated to assess antioxidant activity^33^. A 20 µg/mL plant extract was added to 1 mM DPPH and 4 mL of methanol. The mixture was centrifuged and kept in darkness for 30 min. The absorbance was then determined at 517 nm, and the result was expressed as a percentage of DPPH radical inhibition using the following equation:

$${\text{Free radical inhibition}}(\% ) \, = \, \left[ {{1} - \left( {{\text{absorbance of sample}}/{\text{absorbance of control}}} \right)} \right] \times { 1}00$$

The reference standard was ascorbic acid; thus, the results are expressed as mg ascorbic acid equivalents antioxidant capacity in g of extract (mg AEAC/g extract).

### Measurement of the minimum inhibitory concentration (MIC) and minimum bactericidal concentration (MBC)

One gram of dry sample was dissolved in 100 mL of ethanol. The extraction was performed at room temperature for three days. Filter paper (Whatman No. 1) was used to filter the mixture. The aqueous extract was concentrated in a rotary evaporator at 40 °C. The extraction yield was calculated for the obtained residue.

The 0.5 McFarland standard (1.5 × 10^8^ CFU/mL) of three bacterial pathogens was diluted with the extract at a 1:200 ratio. Fifty microliters of nutrient broth was added. After incubating at 37 °C for 24 h, ten microliters of 0.18% resazurin was added; subsequently, the mixture was incubated for 2 h. The MIC was determined by the minimum bacterial number and calculated following previously described methods^34^.

The MIC was then subcultured on Mueller–Hinton agar plates. To determine the MBC, the mixture was incubated at 37 °C for 24 h. The MBC achieved the lowest concentration associated with the flower extract, killing > 99.9% of the initial bacteria^35^.

### Data analysis

This study included six replicates (n = 6) for plant growth parameters and three replicates (n = 3) for chlorophyll and carotenoid contents, antioxidant enzyme activity, MDA content, phenolic content, flavonoid content, and DPPH radical scavenging capacity. The obtained data are presented as the mean value and standard deviation. One-way analysis of variance (ANOVA) with Duncan's multiple range test^36^ (p < 0.05) was performed to test the significance of the results using SPSS software, version 26.0.

## Results and discussion

###  Characterization of leucoxene mineral

**Figure 1:**
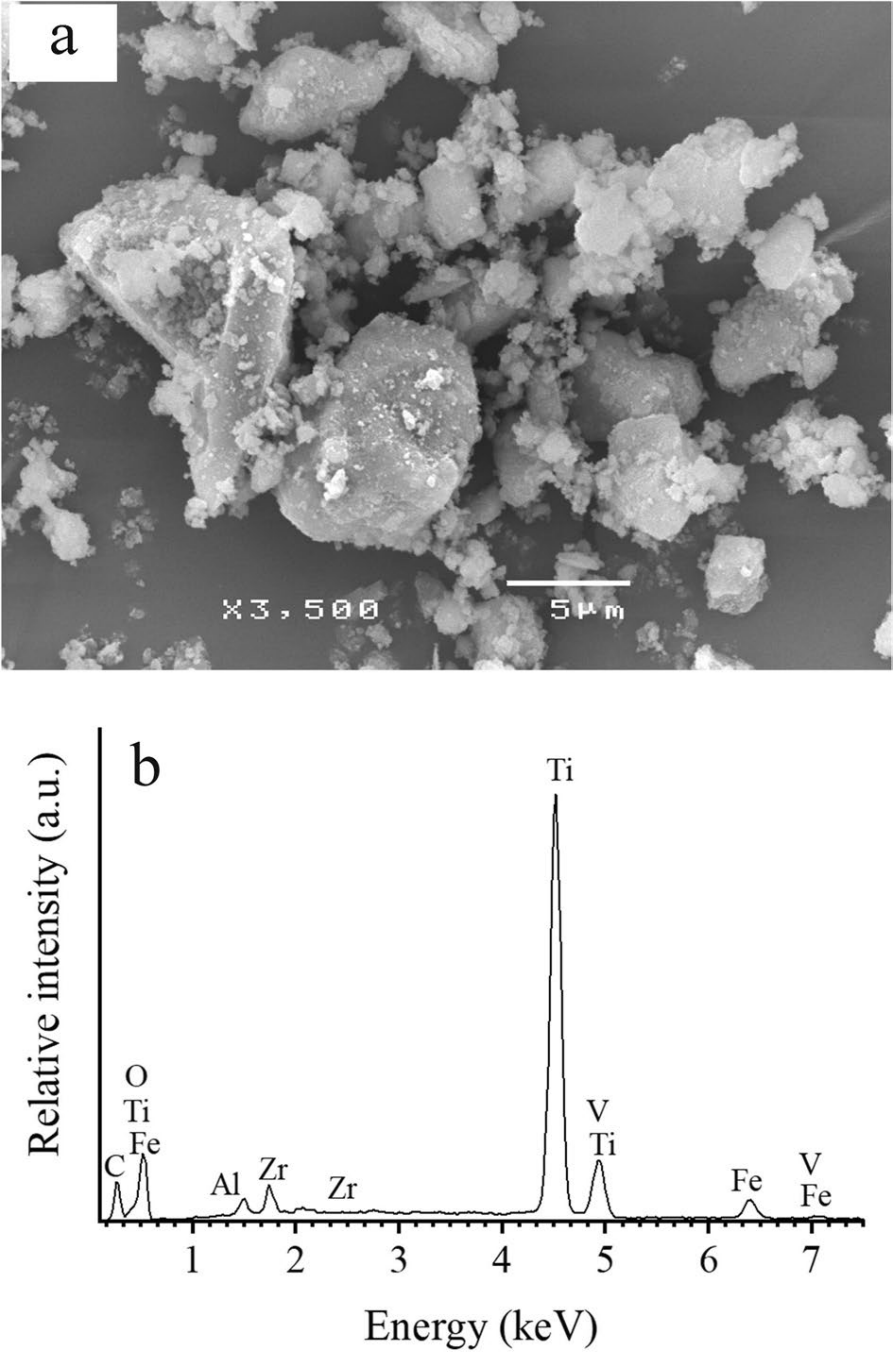
(**a**) SEM image at a magnification of ×3500 and (**b**) EDS spectrum of natural the leucoxene mineral.

**Figure 2:**
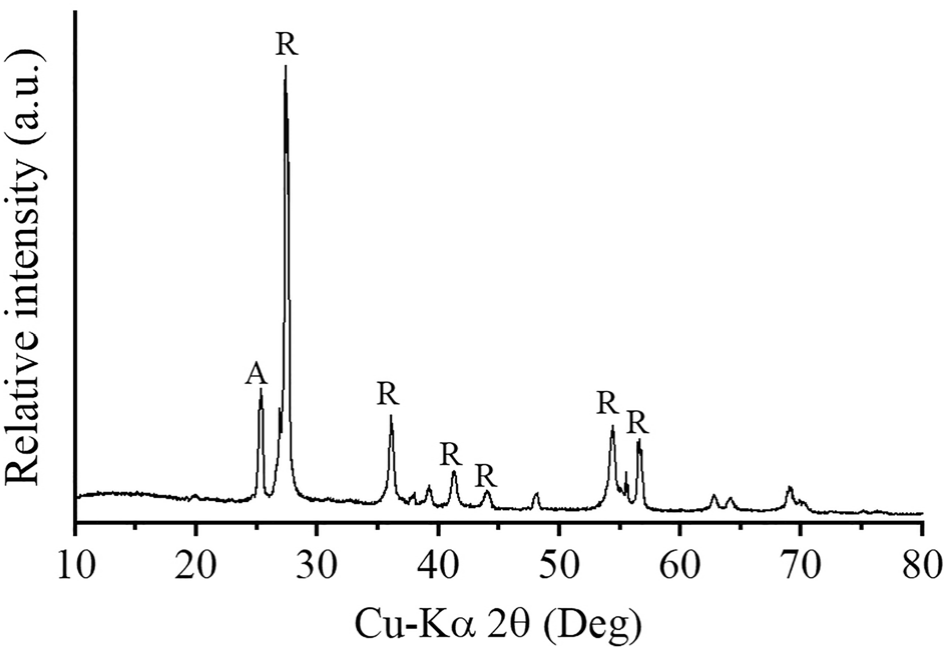
XRD pattern of natural LM. R and A represent the rutile structure and anatase phase, respectively.

To study the morphology, size and composition of LM, SEM and EDS were performed. Figure 1 shows the SEM and EDS images of the natural LM after ball milling at 250 rpm for 12 min. The natural LM is granular, with average particle sizes ranging from 1 to 40 µm (Fig. 1a). Figure 1b presents the EDS spectrum of the natural LM. In the selected area, the EDS spectrum shows a strong signal of the titanium area and ensures the formation of titanium dioxide. Table 1 displays the chemical composition of the natural LM as detected by EDS, which indicates that it consists of 85.97% TiO_2_, 5.82% FeO, 3.82% SiO_2_, 2.06% Al_2_O_3_, 1.37% vanadium oxide (V_2_O_5_), and 0.96% ZrO_2_. Figure 2 shows the XRD pattern of the natural LM, which reveals its crystalline structure. The XRD patterns show strong diffraction peaks at approximately 27°, 36°, 41°, 44° and 64°, indicating that TiO_2_ is present in the rutile (R) structure. In addition, a dominant diffraction peak is also observed at approximately 25°, which indicates that TiO_2_ is in the anatase (A) phase. The EDS and XRD results correspond to those of a previous report^29^. Typically, the micro- or nanosized TiO_2_ containing the anatase and rutile phases has been reported to be an efficient photocatalyst that is able to produce ROS^20^. Hence, increasing concentrations of LM led to a direct increase in plant growth.

**Table 1:** Chemical composition of natural LM.

Composition	LM (%)
TiO_2_	85.97
FeO	5.82
SiO_2_	3.82
Al_2_O_3_	2.06
V_2_O_5_	1.37
ZrO_2_	0.96

### The leucoxene mineral modulates plant growth

Table 2 displays the effect of varying concentrations of LM on the growth of *C. indicum* cuttings in in vitro culture over a period of 8 weeks. The results showed that the height of *C. indicum* plants treated with 40 mg/L LM was significantly greater than that of the control plants at the second and fourth weeks, with increases of 35.71 and 34.49%, respectively. However, there were no significant differences between the control plants and plants treated with 50 or 60 mg/L LM in the second week, and there were no significant differences between the control plants and plants treated with 10, 20, 30, 50, or 60 mg/L LM. In the sixth week, the plant height in response to the 40 mg/L and 50 mg/L LM treatments was significantly greater than that in the control, with increases of 53.64 and 39.80%, respectively. In contrast, plant height in the treatment with 10 mg/L LM decreased slightly (8.53%), but the difference was not significant compared to that in the control. By the eighth week, plant height significantly improved in the 30–60 mg/L LM treatment group, with increases of 25.16, 73.13, 42.34, and 22.47%, respectively, compared to those in the control group. The heights of the plants treated with 10 and 20 mg/L LM did not differ from that of the control plants. The results indicate that the greatest increase in the height of *C. indicum* was observed in response to the 40 mg/L LM treatment.

**Table 2:** Effect of LM on the growth parameters of *C. indicum*.

Growth parameters	LM treatment (mg/L)	Week of cultivation			
		2	4	6	8
Plant height (mm)	Control	3.50 ± 0.66a	8.65 ± 0.93a	15.12 ± 1.45ab	21.22 ± 3.07ab
	10	4.20 ± 0.49abc	8.62 ± 0.62a	13.83 ± 1.16a	20.13 ± 1.42a
	20	4.32 ± 0.73bc	9.35 ± 0.96a	16.50 ± 1.91b	23.73 ± 2.72bc
	30	4.02 ± 0.68abc	9.40 ± 1.29a	16.83 ± 1.36b	26.53 ± 2.25c
	40	4.75 ± 0.31c	11.63 ± 1.65b	23.23 ± 3.09c	36.73 ± 3.62d
	50	3.67 ± 0.60ab	9.87 ± 1.12a	21.13 ± 2.3c	30.20 ± 2.80e
	60	3.52 ± 0.37a	9.45 ± 1.56a	15.19 ± 2.11ab	25.98 ± 2.83c
Root number	Control	1.67 ± 0.82a	2.50 ± 0.84a	2.50 ± 0.84a	2.67 ± 0.82a
	10	1.50 ± 0.55a	2.00 ± 0.89a	2.00 ± 0.63a	2.33 ± 0.82a
	20	2.00 ± 0.63a	2.50 ± 0.55a	2.50 ± 0.84a	2.67 ± 0.82a
	30	1.67 ± 0.82a	1.67 ± 0.82a	2.83 ± 0.41a	3.17 ± 0.41a
	40	1.50 ± 0.55a	2.17 ± 0.41a	2.17 ± 0.41a	2.83 ± 0.98a
	50	1.33 ± 0.82a	2.17 ± 0.98a	2.33 ± 0.82a	3.50 ± 1.22a
	60	1.17 ± 0.41a	1.83 ± 0.75a	2.17 ± 0.41a	2.83 ± 0.98a
Leaf number	Control	3.00 ± 0.63ab	5.83 ± 0.41a	8.17 ± 0.75ab	9.50 ± 0.84a
	10	3.67 ± 0.82bc	6.00 ± 0.63a	7.67 ± 1.03a	9.33 ± 1.03a
	20	3.50 ± 0.55abc	6.00 ± 0.00a	7.67 ± 0.52a	9.50 ± 0.84a
	30	2.83 ± 0.75ab	6.00 ± 0.63a	8.50 ± 1.52ab	10.83 ± 1.72ab
	40	3.67 ± 0.82bc	6.50 ± 0.84a	9.17 ± 1.17ab	12.33 ± 1.86b
	50	4.00 ± 0.63c	6.67 ± 1.03a	9.33 ± 1.75b	11.83 ± 2.48b
	60	2.67 ± 0.82a	5.83 ± 0.98a	9.17 ± 0.98ab	11.50 ± 1.38b
Node number	Control	1.17 ± 0.41a	3.67 ± 0.52abc	5.67 ± 0.52a	6.83 ± 1.17a
	10	1.17 ± 0.41a	3.33 ± 0.82ab	5.67 ± 0.82a	6.83 ± 0.98a
	20	1.67 ± 0.52a	4.33 ± 0.82bc	6.00 ± 0.89ab	7.33 ± 0.82a
	30	1.17 ± 0.52a	4.00 ± 0.63abc	6.33 ± 0.52ab	7.50 ± 1.52a
	40	1.33 ± 0.52a	4.50 ± 1.05c	7.00 ± 1.41b	9.00 ± 1.26b
	50	1.17 ± 0.41a	3.17 ± 1.17a	6.00 ± 0.89ab	8.17 ± 0.75ab
	60	1.50 ± 0.55a	4.17 ± 0.41abc	6.50 ± 0.84ab	7.83 ± 1.47ab

Significantly different values (p < 0.05) are indicated by different letters.

The number of roots of *C. indicum* treated with LM throughout cultivation was not significantly different from that of the untreated plants at every week of cultivation, as shown in Table 2. The leaf number of plants treated with 40 and 50 mg/L LM was significantly greater than that of the control (22.22 and 33.33% increase, respectively) in the second week. No significant differences were detected between the control plants and plants treated with 10, 20, 30, or 60 mg/L LM. An insignificant difference was observed between the untreated and treated groups in the fourth week. In the sixth week, the number of plants treated with 50 mg/L LM was significantly greater than that in the control group (31.25% increase). There were no significant differences between the control and the other conditions. In the final week of cultivation, the leaf number of plants treated with 40–60 mg/L LM was significantly greater than that of untreated plants, with increases of 29.82, 24.56, and 21.05%, respectively. However, no significant difference was detected between the control plants and plants treated with 10, 20, or 30 mg/L LM.

**Figure 3:**
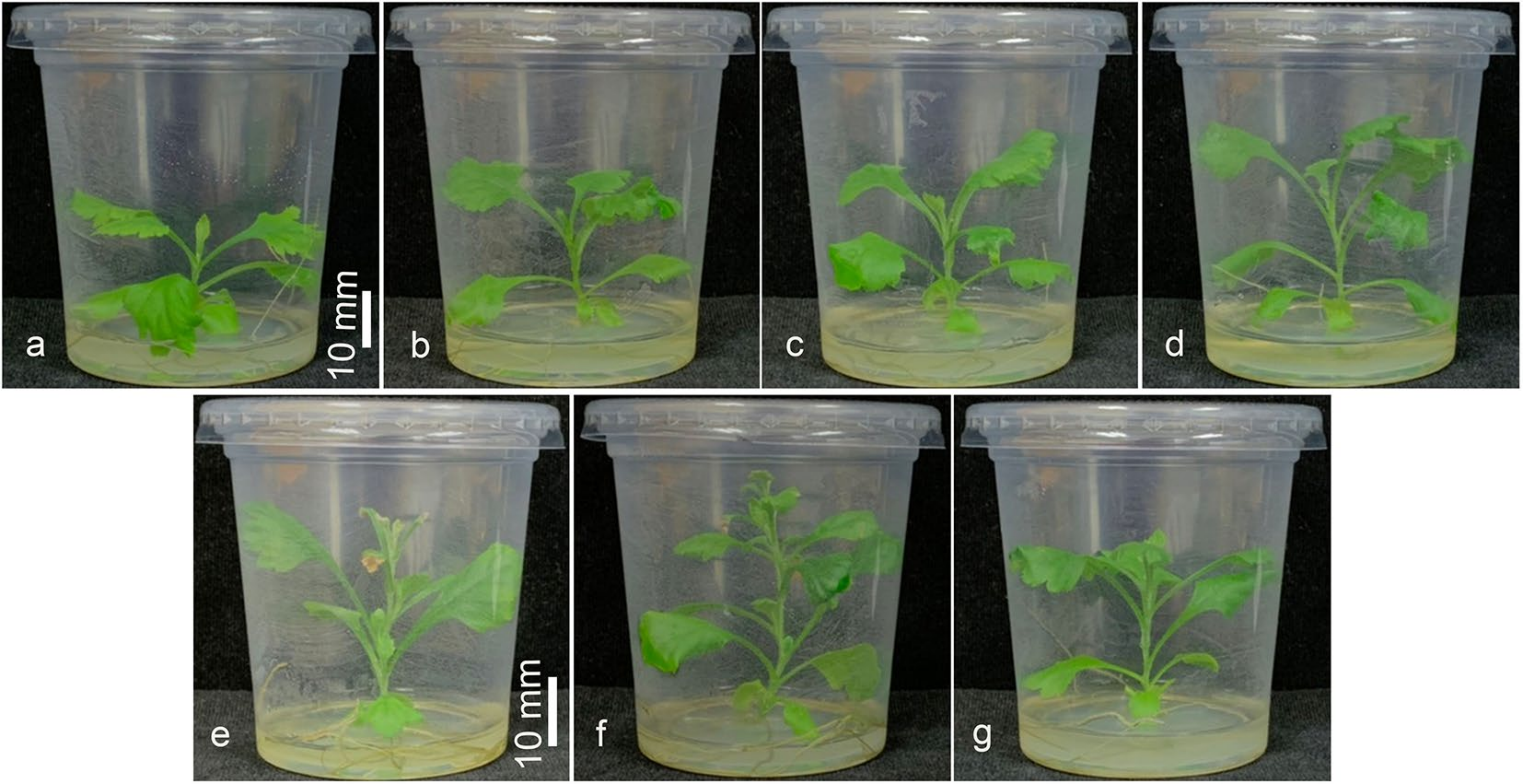
Images of *C. indicum* (**a**) untreated and treated LM at (**b**) 10, (**c**) 20, (**d**) 30, (**e**) 40, (**f**) 50, and (**g**) 60 mg/L after the 8th week of cultivation.

In the second week, the number of nodes did not significantly differ between the untreated and treated groups. In the fourth week, the node number of plants treated with 40 and 60 mg/L LM was significantly greater than that of the control (increases of 22.73 and 13.64%, respectively), whereas there were no significant differences between untreated plants and those treated with 10, 20, 30, or 50 mg/L LM. In the sixth and eighth weeks, the node number of plants exposed to 40 mg/L LM was greater than that of the control plants, with increases of 23.46 and 31.77%, respectively. No significant differences were detected between the control group and the other LM treatment groups. In addition, Fig. 3 shows the agronomy of the plants grown with different concentrations of LM during the eighth week of cultivation. Compared with that in the control treatment, the plant height in the LM treatment group improved.

Table 3 shows the impact of LM on the root length, fresh shoot weight, root fresh weight, shoot dry fresh weight, and root dry weight of *C. indicum* cuttings in vitro after the eighth week of cultivation. The root length ranged from 17.12 to 25.55 mm, with the control group having a significantly greater root length than that in the treated groups. The lengths of the roots of plants treated with 10–60 mg/L LM decreased significantly, by 17.70, 24.98, 18.21, 21.09, 13.37, and 35.53%, respectively.

**Table 3:** Root length, shoot fresh weight, root fresh weight, dry shoot weight, and root dry weight of *C. indicum* cuttings cultured in vitro with LM treatment.

LM treatment (mg/L)	Root length (mm)	Shoot fresh weight (g)	Root fresh weight (g)	Dry shoot weight (g)	Root dry weight (g)
Control	26.55 ± 2.43a	0.167 ± 0.021a	0.125 ± 0.010a	0.0171 ± 0.0032a	0.0063 ± 0.0008a
10	21.85 ± 2.80b	0.143 ± 0.038a	0.083 ± 0.0082b	0.0121 ± 0.0020b	0.0055 ± 0.0011a
20	19.12 ± 2.11bc	0.242 ± 0.025b	0.102 ± 0.010ab	0.0155 ± 0.0042ab	0.0075 ± 0.0013a
30	21.72 ± 3.28b	0.248 ± 0.031b	0.155 ± 0.014ac	0.0245 ± 0.0025c	0.0141 ± 0.0031b
40	20.95 ± 3.81b	0.332 ± 0.015d	0.230 ± 0.019d	0.0317 ± 0.0040d	0.0191 ± 0.0026d
50	23.00 ± 3.79b	0.335 ± 0.024d	0.252 ± 0.066d	0.0271 ± 0.0053c	0.0150 ± 0.0038b
60	17.12 ± 1.34c	0.283 ± 0.029c	0.187 ± 0.020c	0.0253 ± 0.0026c	0.0139 ± 0.0019b

Significantly different values (p < 0.05) are indicated by different letters.

The shoot fresh weight varied from 0.143 to 0.335 g and increased in response to the LM treatments, with the maximum shoot weight recorded at 50 mg/L (increased by 101.10%), followed by 40 mg/L (increased by 99.00%) and 60 mg/L (increased by 70.00%). In contrast, a decrease in shoot fresh weight was observed after applying 10 mg/L LM, decreasing by 14.37% compared to that of the control, but no significant difference was found.

The root fresh weight varied from 0.083 to 0.252 g (Table 3). The fresh weight of the roots also increased in the LM treatment group. The maximum value of root fresh weight was recorded after plants were subjected to 50 mg/L LM treatment (an increase of 101.33%), followed by 40 mg/L LM treatment (an increase of 84.00%) and 60 mg/L LM treatment (an increase of 49.33%). Nevertheless, plants treated with 10 and 30 mg/L LM exhibited decreases in root fresh weight of 33.60% and 18.40%, respectively.

The shoot dry weight ranged from 0.0121 to 0.0317 g (Table 3). The maximum shoot dry weight was observed in the 40 mg/L LM treatment group (85.27%), followed by the 50 mg/L LM treatment (58.54%) and the 60 mg/L LM treatment (47.80%) groups. A negative effect on shoot dry weight was recorded in the 10 and 20 mg/L LM treatments, which decreased by 29.24 and 9.36%, respectively, compared with that in the control. A significant difference was found in the 10 mg/LM treatment group, but no significant differences were observed in the 20 mg/L LM treatment group compared to the control group.

The root dry weight ranged from 0.0055 to 0.0191 g (Table 3). The root dry weight significantly increased with the addition of 30–60 mg/L LM. Notably, the maximum root dry weight was recorded in the 40 mg/L LM treatment (increased by 202.11%), followed by the 50 mg/L LM treatment (increased by 136.05%) and the 30 mg/L LM treatment (increased by 184.21%). There were significant differences between the control and plants treated with 10 and 20 mg/L LM. However, a decrease in root dry weight was found in plants treated with 10 mg/L LM (a decrease of 12.30%) compared to the control.

### Chlorophyll and total carotenoid contents

The results showed that different concentrations of LM significantly impacted the *Chl* a, *Chl* b, total chlorophyll, and total carotenoid contents (Table 4). The results showed that the content of *Chl* a in the plants treated with 30–60 mg/L LM was significantly greater than that in the control plants, increasing by 37.50, 47.12, 42.31, and 20.19%, respectively. No significant difference was detected between untreated plants and plants treated with 10 or 20 mg/L LM.

**Table 4:** Photosynthetic pigments of *C. indicum* cuttings cultured in vitro under LM treatment.

LM treatment (mg/L)	*Chl* *a* (mg/g DW)	*Chl* *b* (mg/g DW)	Total chlorophyll (mg/g DW)	Total carotenoids (mg/g DW)
Control	1.04 ± 0.05^c^	0.61 ± 0.01^d^	1.65 ± 0.03f.	0.26 ± 0.00^b^
10	1.03 ± 0.04^c^	0.74 ± 0.01^c^	1.77 ± 0.04^e^	0.26 ± 0.01^b^
20	1.08 ± 0.03^c^	0.64 ± 0.02^d^	1.72 ± 0.01^e^	0.25 ± 0.00^b^
30	1.43 ± 0.04^a^	0.75 ± 0.02^c^	2.18 ± 0.02^c^	0.31 ± 0.01^a^
40	1.53 ± 0.04^a^	1.11 ± 0.02^a^	2.64 ± 0.06^a^	0.30 ± 0.00^a^
50	1.48 ± 0.14^a^	0.81 ± 0.09^bc^	2.29 ± 0.05^b^	0.31 ± 0.01^a^
60	1.25 ± 0.02^b^	0.83 ± 0.04^b^	2.08 ± 0.04^d^	0.25 ± 0.01^b^

Significantly different values (p < 0.05) are indicated by different letters.

Plants treated with all LM concentrations exhibited greater *Chl* b contents than did the control plants. The highest *Chl* b content was detected in the 40 mg/L LM-treated plants (an increase of 81.97%), followed by 60 mg/L LM-treated plants (an increase of 36.07%) and 50 mg/L LM-treated plants (an increase of 32.79%). The *Chl* b content of plants treated with 20 mg/L LM did not significantly differ from that of the control group.

Compared with those of the control plants, the total chlorophyll content of the plants under all LM conditions significantly increased. The maximum total chlorophyll content was recorded in the 40 mg/L LM treatment (60.00%), followed by the 50 mg/L LM treatment (38.79%) and the 30 mg/L LM treatment (32.12%).

The total carotenoid content ranged from 0.26 to 0.31 mg/g DW, as shown in Table 4. The 30 and 50 mg/L LM treatments had the greatest total carotenoid content (0.31 mg/L), followed by the 40 mg/L LM treatment, while an insignificant difference was found between the control plants and those treated with 10 and 20 LM.

### Lipid peroxidation and enzyme activities

**Figure 4:**
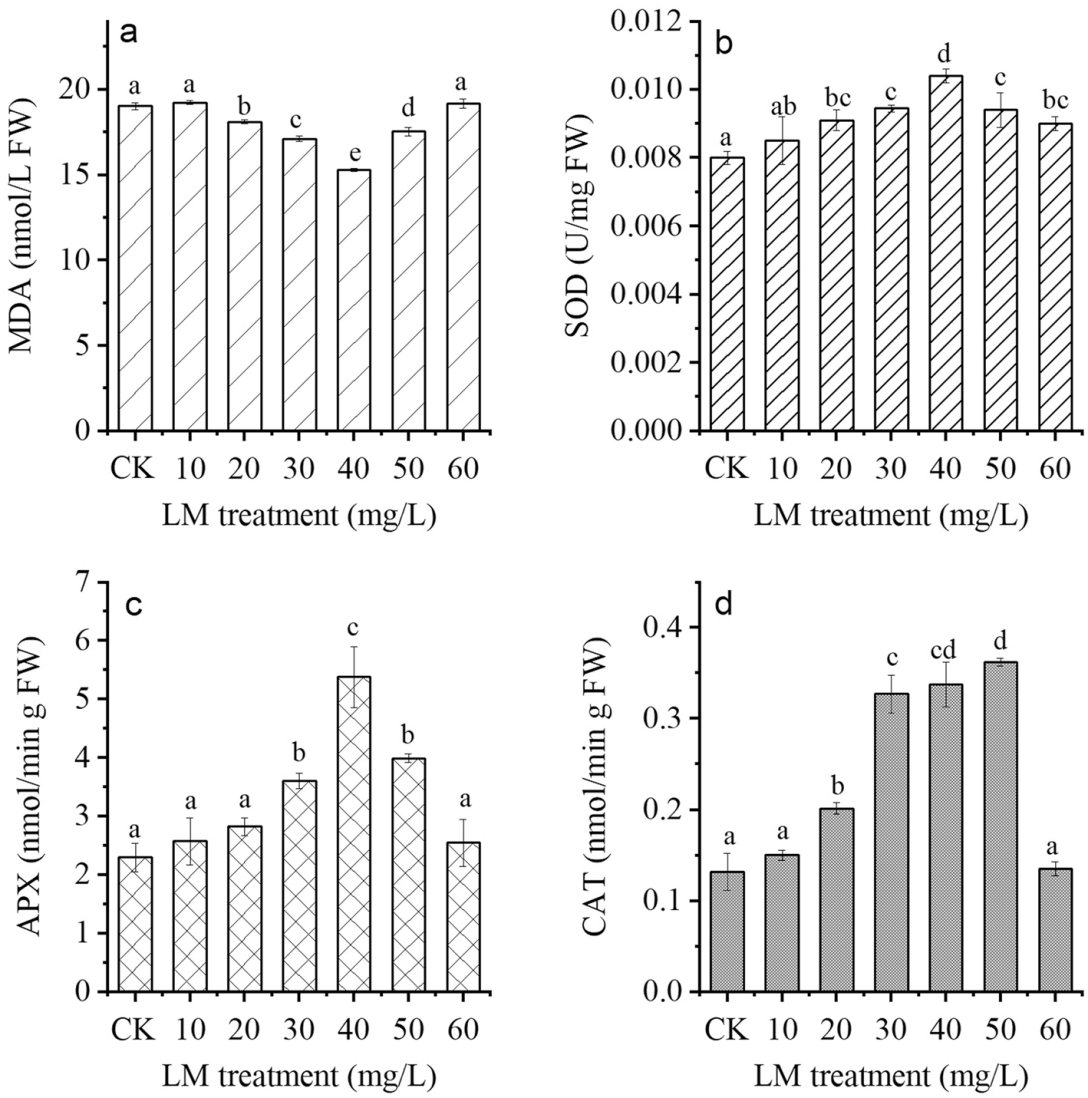
Contents of (**a**) malondialdehyde (MDA), (**b**) superoxide dismutase (SOD), (**c**) ascorbate peroxidase (APX), and (**d**) catalase (CAT) in *C*. *indicum* under six LM conditions (10, 20, 30, 40, 50, and 60 mg/L) after eight weeks of cultivation. CK represents the control group. The results are expressed as the mean ± standard error bar (n = 3). Significantly different values (p < 0.05) are indicated by different letters.

The changes in lipid peroxidation and enzyme activities in plant leaves after LM treatment are depicted in Fig. 4. The MDA level in the plants ranged from 15.30 to 19.25 nM/g FW (Fig. 4a). Compared with the control treatment, the 20–40 mg/L LM treatment reduced the MDA content, while the 10 and 60 mg/L LM treatments had no significant effect on the MDA content. The 40 mg/L LM treatment had the lowest MDA content (a reduction of 19.58%), followed by the 50 mg/L LM treatment (a reduction of 10.04%).

SOD activity in *C. indicum* markedly increased in all LM treatments, increasing by 6.25–30%, as shown in Fig. 4b. The 40 mg/L LM treatment had the highest SOD activity (an increase of 30%), followed by the 30 (an increase of 18.13%) and 50 mg/L (an increase of 18.13%) leucoxene treatments. The SOD activity of plants treated with 10 mg/L LM did not significantly differ from that of the control group.

APX activity in plants treated with 30–50 mg/L LM was significantly greater than that in the control (Fig. 4c). The maximum level of APX activity was found in response to the 40 mg/L LM treatment, followed by 50 and 30 mg/L LM treatments, which was approximately 133.91, 73.91, and 56.52% compared to the control, respectively. There were no significant differences between control plants and plants treated with 10, 20, or 60 mg/L LM.

Similarly, the activity of CAT in *C. indicum* treated with LM was greater than that in the control (Fig. 4d). The maximum CAT activity was found in response to the 60 mg/L LM treatment (an increase of approximately threefold), followed by the 40 and 30 mg/L LM treatments (an increase of approximately 2.5-fold). No significant difference was detected between untreated plants and plants treated with 10 or 60 mg/L LM.

### Bioactive compounds

**Figure 5:**
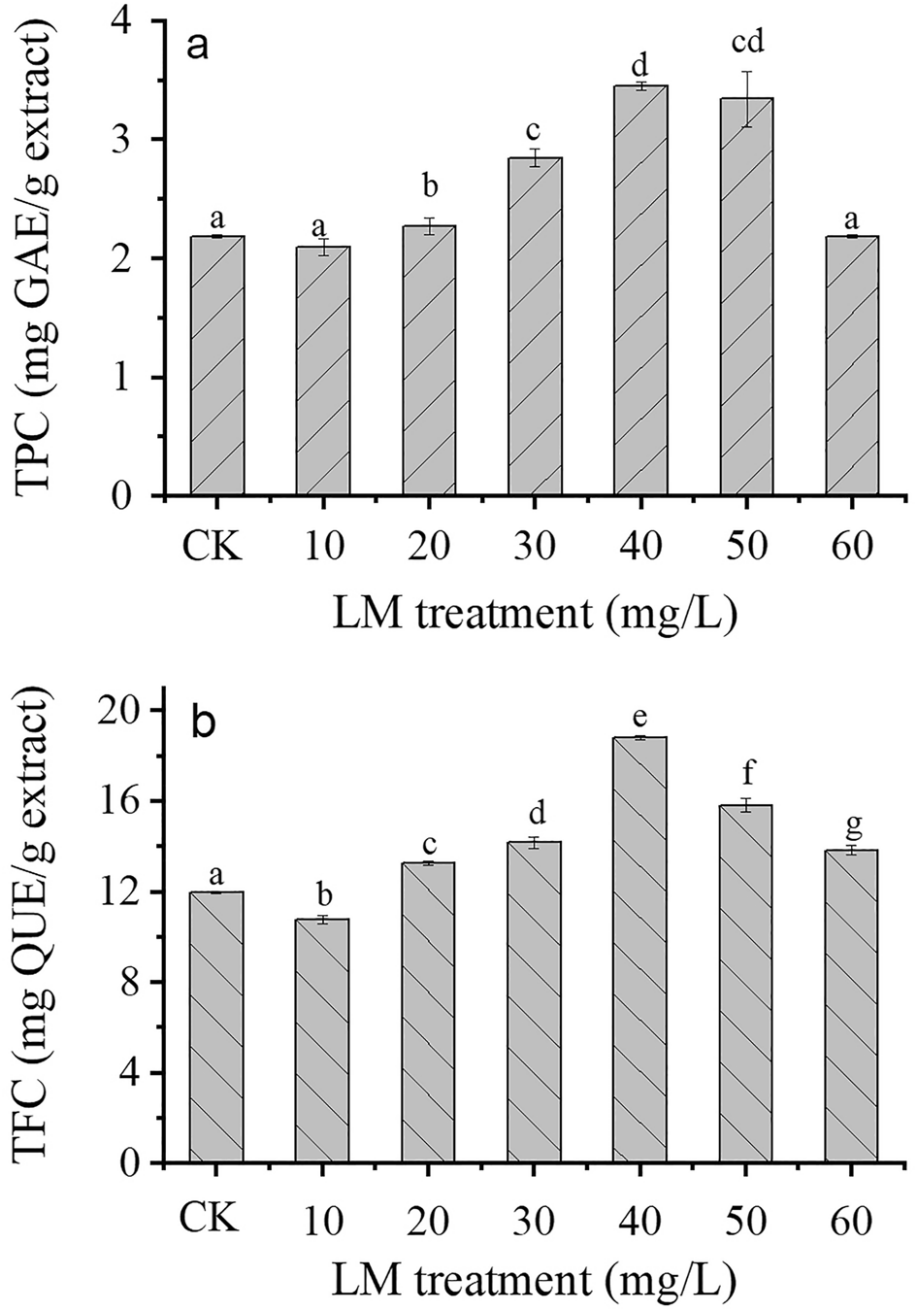
(**a**) Total phenolic content (TPC) and (**b**) total flavonoid content (TFC) in *C*. *indicum* treated with LM at six concentrations (10, 20, 30, 40, 50, and 60 mg/L) after 8 weeks of cultivation. CK represents the control group. The results are expressed as the mean ± standard error bar (n = 3). Significantly different values (p < 0.05) are indicated by different letters.

The total phenolic content (TPC) and total flavonoid content (TFC) of treated and control *C*. *indicum* are summarized in Fig. 5. The TPC of *C*. *indicum* treated with LM varied from 2.09 to 3.45 mg GAE/g extract, while the control contained 2.19 mg GAE/g extract (Fig. 5a). The TPC showed positive changes when 30–50 mg/L LM was applied. The maximum TPC occurred at 40 mg/L LM treatment (an increase of 57.65%), followed by 50 mg/L LM treatment (an increase of 57.28%) and 30 mg/L LM treatment (an increase of 30.20%). However, nonsignificant differences between the control and 10 and 60 mg/L LM treatment were observed.

The TFC of *C*. *indicum* treated with LM ranged from 10.59 to 18.73 mg QUE/g extract, while that of the control was 11.95 mg QUE/g extract, as depicted in Fig. 5b. The LM treatments (30–60 mg/L) significantly improved the TFC (Fig. 5b). The 40 mg/L LM treatment had the greatest increase in TFC (57.23%), followed by the 50 mg/L (32.14%) and 30 mg/L LM (18.40%) treatments. Conversely, a significant reduction in TFC of approximately 10% was observed in the 10 mg/L LM treatment compared to the control.

### Antioxidant activity

**Figure 6:**
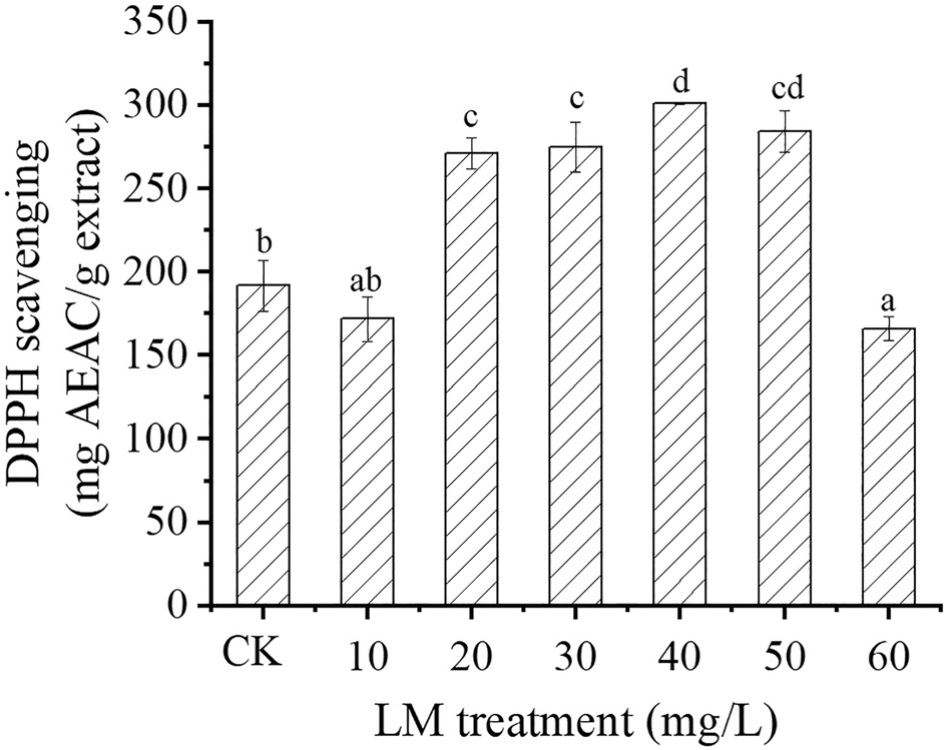
Antioxidant activity (DPPH scavenging activity) in *C*. *indicum* treated with LM at six concentrations (10, 20, 30, 40, 50, and 60 mg/L) after 8 weeks of cultivation. The results are expressed as the mean ± standard error bar (n = 3). CK represents the control group. Significantly different values (p < 0.05) are indicated by different letters.

The change in the DPPH free radical scavenging capacity of *C. indicum* grown under LM treatment and the untreated control is shown in Fig. 6. The DPPH scavenging activity in *C*. *indicum* grown with LM (20–50 mg/L) was greater than that in the untreated group. The highest DPPH scavenging activity was obtained in the 40 mg/g LM treatment group (an increase of 56.97%), followed by the 30 mg/L LM treatment (an increase of 29.19%) and the 20 and 50 mg/L LM treatments (increases of 41.39 and 43.34%, respectively) groups. However, a significant reduction in DPPH scavenging activity, by 10.43 and 13.46%, was observed in the 10 and 60 mg/L LM treatments, respectively, compared to the control. In addition, the coefficient of variation (CV) analysis of all results is shown in the Supplementary file.

### Minimum inhibitory concentration (MIC) and minimum bactericidal concentration (MBC) of *C*. *indicum*

Table 5 displays the MIC and MBC values of the *C. indicum* extract against the three bacterial pathogens. The aqueous leaf extract of *C. indicum* grown under LM conditions and the control had the MIC, < 3.125 mg/mL, against *Candida albicans*, with a MBC < 3.125 mg/mL. The MIC and MBC values for *Staphylococcus aureus* and *Escherichia coli* were < 3.125 mg/mL for *C. indicum* grown under the 40 and 50 mg/L LM treatments. In addition, the 30 mg/L treatment effectively inhibited *Staphylococcus aureus,* with MIC and MBC values (< 3.125 mg/mL). These results indicate that plants grown with different concentrations of LM significantly inhibited the growth of bacterial pathogens.

**Table 5:** MIC and MBC values of *C*. *indicum* grown under different concentrations of LM against bacterial pathogens.

LM treatment (mg/L)	*Staphylococcus aureus*		*Escherichia coli*		*Candida albicans*	
	MIC (mg/mL)	MBC (mg/mL)	MIC (mg/mL)	MBC (mg/mL)	MIC (mg/mL)	MBC (mg/mL)
CK	25	50	50	100	< 3.125	< 3.125
10	50	100	50	100	< 3.125	< 3.125
20	25	50	50	100	< 3.125	< 3.125
30	< 3.125	< 3.125	50	100	< 3.125	< 3.125
40	< 3.125	< 3.125	< 3.125	< 3.125	< 3.125	< 3.125
50	< 3.125	< 3.125	< 3.125	< 3.125	< 3.125	< 3.125
60	25	50	50	100	< 3.125	< 3.125

## Discussion

In this investigation, a low-cost natural LM was applied to *C*. *indicum* to understand its effects on medicinal plants. The chemical composition of the natural LM revealed by EDS indicated that the natural LM was composed of ~ 86% TiO_2_. The XRD results identified the main phase of anatase and rutile TiO_2_ with band gaps of 3.2 and 3.0 eV, respectively. The mixed-phase crystallinity of both phases significantly reduces the recombination of electron–hole pairs. According to Nosaka et al.^37^, photoelectrons preferentially excite the conduction band of rutile because anatase has a lower valence edge than rutile. Holes generated from rutile can quickly pass to the anatase phase and are associated with ROS production. These processes could reduce the recombination of electron–hole pairs^37^. Consequently, additional ROS can be produced.

According to our results, we found that LM improved the physiological and biochemical parameters of *C*. *indicum* plants. The positive impacts on *C*. *indicum* growth (especially on plant height, shoot fresh and dry weights, and root fresh and dry weights) were significant in response to the LM treatments at concentrations ranging from 30–50 mg/L, as depicted in Tables 2 and 3. A previous study showed that 100–200 mg/L TiO_2_ enhanced the growth of tomato and onion plants (shoot and root lengths and shoot fresh and dry weights)^38^. Similarly, 50–100 mg/L TiO_2_ improved the leaf number, leaf fresh weight, and dry weight of *D. moldavica*^21^. In addition, improvements in plant growth parameters were observed after FeO, SiO_2_, and Al_2_O_3_ exposure^39^.

The increase in plant growth might be correlated with the increase in photosynthesis, as indicated by the increase in chlorophyll and carotenoid contents. An increase in chlorophyll and carotenoid contents is conducive to improving photosynthesis efficiency, enhancing the synthesis of carbohydrates and increasing the fresh and dry weights of plants^23^. ROS play dual roles in chloroplast function. First, they are a byproduct of the photosynthetic electron transport chain, and their accumulation can cause oxidative damage to various components of the chloroplast, including chlorophyll and carotenoids^40,41^. However, moderate levels of ROS have been shown to stimulate chlorophyll biosynthesis and enhance chloroplast development. Various studies have also demonstrated that ROS act as signaling molecules in regulating chlorophyll biosynthesis. In particular, singlet oxygen (^1^O_2_) has been shown to stimulate the expression of genes involved in chlorophyll biosynthesis and promote chlorophyll accumulation in the chloroplast^40^. This effect is thought to be mediated by the activation of transcription factors regulating chlorophyll biosynthesis-related gene expression^40^. A previous study reported that TiO_2_ promoted chlorophyll and carotenoid contents in radish^23^, peppermint^42^, and maize^43^. Furthermore, the growth factors of *D. moldavica* plants treated with 100 mg/L TiO_2_ improved with increasing levels of *Chl* a, *Chl* b, and total carotenoids. On the other hand, a higher concentration of TiO_2_ could damage plant growth and photosynthetic pigments^20,21^. In the present study, compared with the 50 mg/L LM treatment, the 60 mg/L LM treatment gradually reduced plant growth. Despite this reduction, the plant growth parameters were not below those of the control, and there was no toxicity in plants treated with LM. In addition, other metal oxides, i.e., FeO, SiO_2_, and Al_2_O_3_, enhance chlorophyll and carotenoid contents in plants^44^.

ROS are generally generated in chloroplasts via intricate and diverse defense systems that enable photosynthesis to operate efficiently even under challenging environmental conditions with limited resources^45^. ROS can participate in oxidative signaling and environmental sensing, which extends their functions associated with regulating photosynthesis. Therefore, ROS accumulation involves many critical plant processes, such as cell-to-cell communication and the control of growth and stress responses^46,47^. However, the overproduction of ROS acts as a signal and plays a key role in oxidative stress, resulting in damage to the cellular components of plants and programmed cell death^48^. Thus, chloroplasts remove excess ROS to prevent oxidative damage in plants^49^ and extend the photosynthetic time of chloroplasts by promoting the activity of antioxidant enzymes, i.e., SOD, CAT, and APX^50^. SOD plays a key role in providing defense against oxidative stress^51^ by activating the dismutation of superoxide radicals (${\text{O}}_{2}^{\cdot - }$) into hydrogen peroxide (H_2_O_2_) and oxygen (O_2_). CAT activity is an essential antioxidant enzyme for the conversion of H_2_O_2_ into water and oxygen. APX is another important enzyme that scavenges H_2_O_2_ to protect chloroplasts and other cells from H_2_O_2_ damage. Therefore, improving antioxidant enzyme activity is strongly associated with high defense against oxidative stress. Notably, enzymatic antioxidants are activated by the formation of ROS during oxidative stress in plants^52^. Our findings demonstrated that LM enhances antioxidant enzyme activities and contributes to effective *C*. *indicum* defense against oxidative stress. In this context, concentrations of LM (30–50 mg/L) promoted increased activity of enzymatic antioxidants (SOD, CAT, and APX), which coincided with reduced levels of MDA, a marker of oxidative damage (Fig. 4). Thus, biochemical profiling of *C*. *indicum* revealed that, compared with those in the control plants, ROS accumulation can stimulate the antioxidant machinery, increase the synthesis of phytohormones, and cause minor oxidative damage in plants grown under LM. Similar to our investigations, increasing enzymatic antioxidants were associated with decreased MDA contents in broad bean plants treated with TiO_2_^20^. Moreover, TiO_2_ enhanced the growth parameters of *D. moldavica* plants by increasing enzyme activity and decreasing the MDA content^21^. Previous evidence has shown the reasonable induction of enzymatic antioxidants after treatment with TiO_2_, thus reducing plant damage related to ROS accumulation^53^. SOD, APX, and CAT activities in plants treated with TiO_2_ have been shown to increase^21,54^. In addition, promoting APX, CAT, glutathione peroxidase (GPX), and SOD activities in spinach plants treated with TiO_2_ can alleviate oxidative damage^55^. A moderate level of TiO_2_ in *Hyoscyamus niger* L. plants increased the CAT, GPX, and APX activities^56^.

Phenolic compounds are a significant group of secondary metabolites in plants that contain at least one aromatic ring bearing one or more hydroxyl groups in their structure. The main structures of these compounds are characterized into three groups: flavonoids, nonflavonoids, and phenolic acids^57,58^. Phenolic compounds exhibit several plant activities, such as scavenging free radicals, providing protection against ultraviolet (UV), biotic or abiotic stress, and combating pathogenic bacteria. An increase in phenolic compounds scavenges ROS and prevents plant damage. In the present study, the 30–50 LM treatments had higher levels of phenolic compounds in the *C*. *indicum* extract than in the control extract (Fig. 5). These results are consistent with those of TiO_2_ NP exposure in wheat plants infected with *Puccinia striiformis*, which led to changes in the TPC and TFC of the plants^59^. Similar findings demonstrated that the TPC and TFC in *Dracocephalum kotschyi* were improved by TiO_2_ NPs and exposure time^54^. Thus, our results show that the TPC and TFC of *C*. *indicum* improved in response to LM treatment. In addition, the content of phenolic compounds in plants may increase in response to other stress conditions, i.e., high temperature and UV light. The phenolic compounds of *C*. *indicum* depend on various factors, i.e., vegetative propagation, cultivation conditions, and soil type^60^.

The current investigation demonstrated that the antioxidant capacity of *C*. *indicum* extract can be optimized and improved by using low-cost LM. The significant change in antioxidants in *C*. *indicum* may be due to its high phenolic compound content. Thus, Pearson’s correlation was performed to determine the relationships between TPC, TFC, and DPPH radical scavenging activity. The experimental evidence showed that DPPH had a strong linear correlation (R = 0.955) with the TPC and a strong correlation (R = 0.740) with the TFC. The results of this study could imply that DPPH in *C*. *indicum* is a function of phenolic compounds, especially TPC and TFC. This study is similar to previous findings that revealed a strong correlation between DPPH radical scavenging activity and the TPC and TFC in mushrooms^33,61^.

The antibacterial properties of aqueous leaf extracts of *C*. *indicum* grown under different LM treatment conditions were evaluated. The lowest MIC and MBC were observed for gram-positive bacteria (*Staphylococcus aureus*), gram-negative bacteria (*Escherichia coli*), and fungi (*Candida albicans*) in response to the 40 and 50 mg/L LM treatments, as shown in Table 5. The highest antibacterial effect is primarily related to phenolic compounds and their correlation with the presence of hydroxyl groups. Indeed, the position and number of these hydroxyl groups in the phenolic ring significantly influence the target bacteria, change the structure of the cell membrane, reduce lipid levels, and suppress bacterial growth^62^. Various reports have shown that phenolic compounds extracted from different fruits can effectively inhibit *Staphylococcus aureus* and *Escherichia coli*^63^. This study suggested that higher levels of phenolic compounds in *C. indicum* grown under LM treatments provide optimal conditions for antibacterial activity. In addition, the key constituents of *C. indium* essential oils, in addition to phenolic compounds such as borneol, camphor,* p*-cymene, and camphene, can also inhibit bacterial growth^5^.

## Conclusion

In the present study, the effect of Thai LM on the growth of *C*. *indicum* cuttings in vitro was evaluated. The LM in this study has a rutile and anatase structure, with a granular shape and grain size of 1–40 µm and a chemical composition of ~ 86% TiO_2_. The LM treatments resulted in greater increases in growth parameters, including plant height, root number, bud number, and fresh and dry weight. LM treatments also increased the chlorophyll and carotenoid contents and antioxidant enzyme activities while decreasing the MDA content, which supported plant growth. The increased phenolic compound content in plants treated with LM has implications for antibacterial applications. The best condition for inhibiting the activity of *Staphylococcus aureus*, *Escherichia coli*, and *Candida albicans* was LM at a concentration of 40–50 mg/L, which yielded the lowest MIC and MBC values. In conclusion, LM with 86% TiO_2_ is a sustainable and cost-effective method for improving *Chrysanthemum* growth-promoting molecules and antibacterial activity, leading to the production of innovative, high-quality *Chrysanthemum* plants.

### Supplementary Information

Supplementary Tables.

## Data Availability

The datasets used and/or analysed during the current study available from the corresponding author on reasonable request.
