# Supplementary material for: Influence of low-cost Thai leucoxene minerals on the growth, bioactive compounds, and antibacterial activities of Chrysanthemum indium L. cuttings in in vitro culture
Source: Sci Rep. 2024 Apr 25;14:9505. doi: 10.1038/s41598-024-60131-5 (PMC11045765; doi:10.1038/s41598-024-60131-5)
Supplement: Supplementary file 1 — Supplementary Tables. [file 41598_2024_60131_MOESM1_ESM.docx]

**Supplementary materials**

**Table S1.** The coefficient of variation (CV) analysis of plant height and root number.

| LM treatment (mg/L) | Plant height (mm) | | | | Root number | | | |
| --- | --- | --- | --- | --- | --- | --- | --- | --- |
|  | 2^nd^ week | 4^th^ week | 6^th^ week | 8^th^ week | 2^nd^ week | 4^th^ week | 6^th^ week | 8^th^ week |
| Control | 3.50 | 8.65 | 15.12 | 21.22 | 1.67 | 2.50 | 2.50 | 2.67 |
| 10 | 4.20 | 8.62 | 13.83 | 20.13 | 1.50 | 2.00 | 2.00 | 2.33 |
| 20 | 4.32 | 9.35 | 16.50 | 23.73 | 2.00 | 2.50 | 2.50 | 2.67 |
| 30 | 4.02 | 9.40 | 16.83 | 26.53 | 1.67 | 1.67 | 2.83 | 3.17 |
| 40 | 4.75 | 11.63 | 23.23 | 36.73 | 1.50 | 2.17 | 2.17 | 2.83 |
| 50 | 3.67 | 9.87 | 21.13 | 30.20 | 1.33 | 2.17 | 2.33 | 3.50 |
| 60 | 3.52 | 9.45 | 15.19 | 25.98 | 1.17 | 1.83 | 2.17 | 2.83 |
| S.D. | 0.47 | 1.02 | 3.46 | 5.70 | 0.27 | 0.31 | 0.28 | 0.38 |
| Average | 4.00 | 9.57 | 17.40 | 26.36 | 1.55 | 2.12 | 2.36 | 2.86 |
| CV(%) | 11.64 | 10.62 | 19.89 | 21.61 | 17.27 | 14.86 | 11.85 | 13.23 |

| LM treatment (mg/L) | Leaf number | | | | Node number. | | | |
| --- | --- | --- | --- | --- | --- | --- | --- | --- |
|  | 2^nd^ week | 4^th^ week | 6^th^ week | 8^th^ week | 2^nd^ week | 4^th^ week | 6^th^ week | 8^th^ week |
| Control | 3.00 | 5.83 | 8.17 | 9.50 | 1.17 | 3.67 | 5.67 | 6.83 |
| 10 | 3.67 | 6.00 | 7.67 | 9.33 | 1.17 | 3.33 | 5.67 | 6.83 |
| 20 | 3.50 | 6.00 | 7.67 | 9.50 | 1.67 | 4.33 | 6.00 | 7.33 |
| 30 | 2.83 | 6.00 | 8.50 | 10.83 | 1.17 | 4.00 | 6.33 | 7.50 |
| 40 | 3.67 | 6.50 | 9.17 | 12.33 | 1.33 | 4.50 | 7.00 | 9.00 |
| 50 | 4.00 | 6.67 | 9.33 | 11.83 | 1.17 | 3.17 | 6.00 | 8.17 |
| 60 | 2.67 | 5.83 | 9.17 | 11.50 | 1.50 | 4.17 | 6.50 | 7.83 |
| S.D. | 0.50 | 0.33 | 0.72 | 1.25 | 0.20 | 0.51 | 0.48 | 0.77 |
| Average | 3.33 | 6.12 | 8.52 | 10.69 | 1.31 | 3.88 | 6.17 | 7.64 |
| CV(%) | 15.00 | 5.38 | 8.40 | 11.68 | 15.46 | 13.05 | 7.80 | 10.11 |

**Table S2.** The coefficient of variation (CV) analysis of leaf number and node number.

**Table S3.** The coefficient of variation (CV) analysis of shoot fresh weight, root fresh weight, root length, dry shoot weight, and root dry weight.

| LM treatment (mg/L) | Shoot fresh weight (g) | Root fresh weight (g) | Root length (mm) | Dry shoot weight (g) | Root dry weight (g) |
| --- | --- | --- | --- | --- | --- |
| Control | 0.167 | 0.125 | 26.55 | 0.0171 | 0.0063 |
| 10 | 0.143 | 0.083 | 21.85 | 0.0121 | 0.0055 |
| 20 | 0.242 | 0.102 | 19.92 | 0.0155 | 0.0075 |
| 30 | 0.248 | 0.155 | 21.72 | 0.0245 | 0.0141 |
| 40 | 0.332 | 0.230 | 20.95 | 0.0317 | 0.0191 |
| 50 | 0.335 | 0.252 | 23.00 | 0.0271 | 0.0150 |
| 60 | 0.283 | 0.187 | 17.12 | 0.0253 | 0.0139 |
| S.D. | 0.075 | 0.064 | 2.88 | 0.0071 | 0.0052 |
| Average | 0.2498 | 0.1619 | 21.5857 | 0.0219 | 0.0116 |
| CV(%) | 29.8760 | 39.4836 | 13.3592 | 32.3408 | 44.6081 |

**Table S4.** The coefficient of variation (CV) analysis of photosynthetic pigments.

| LM treatment (mg/L) | *Chl* a  (mg/g DW) | *Chl* b  (mg/g DW) | Total chlorophyll (mg/g DW) | Total carotenoids (mg/g DW) |
| --- | --- | --- | --- | --- |
| Control | 1.04 | 0.61 | 1.65 | 0.26 |
| 10 | 1.03 | 0.74 | 1.77 | 0.26 |
| 20 | 1.08 | 0.64 | 1.72 | 0.25 |
| 30 | 1.43 | 0.75 | 2.18 | 0.31 |
| 40 | 1.53 | 1.11 | 2.64 | 0.30 |
| 50 | 1.48 | 0.81 | 2.29 | 0.31 |
| 60 | 1.25 | 0.83 | 2.08 | 0.25 |
| S.D. | 0.22 | 0.16 | 0.36 | 0.03 |
| Average | 1.26 | 0.78 | 2.05 | 0.28 |
| CV(%) | 17.23 | 21.03 | 17.51 | 10.14 |

**Table S5.** The coefficient of variation (CV) analysis of malondialdehyde (MDA), superoxide dismutase (SOD), ascorbate peroxidase (APX), and catalase (CAT) activities.

| LM treatment (mg/L) | MDA  (nmol/L FW) | SOD  (U/mg FW) | APX (nmol/min g FW) | CAT (nmol/min g FW) |
| --- | --- | --- | --- | --- |
| Control | 19.03 | 0.0080 | 2.30 | 0.13 |
| 10 | 19.25 | 0.0085 | 2.58 | 0.15 |
| 20 | 18.11 | 0.0091 | 2.82 | 0.20 |
| 30 | 17.12 | 0.0095 | 3.60 | 0.33 |
| 40 | 15.30 | 0.0104 | 5.38 | 0.34 |
| 50 | 17.54 | 0.0094 | 3.99 | 0.36 |
| 60 | 19.17 | 0.0090 | 2.55 | 0.14 |
| S.D. | 1.43 | 0.0008 | 1.10 | 0.10 |
| Average | 17.93 | 0.0091 | 3.32 | 0.24 |
| CV(%) | 7.96 | 8.3485 | 33.07 | 43.99 |

**Table S6.** The coefficient of variation (CV) analysis of total phenolic content (TPC) and total flavonoid content (TFC), and DPPH scavenging activity

| LM treatment (mg/L) | TPC  (mg GAE/g extract) | TFC  (mg QUE/g extract) | DPPH  (mg AEAC/g extract) |
| --- | --- | --- | --- |
| Control | 2.19 | 11.9740 | 191.86 |
| 10 | 2.09 | 10.7700 | 171.89 |
| 20 | 2.85 | 13.2660 | 271.27 |
| 30 | 3.16 | 14.1770 | 284.33 |
| 40 | 3.45 | 18.8270 | 301.17 |
| 50 | 3.34 | 15.8227 | 275.01 |
| 60 | 2.27 | 13.8450 | 166.06 |
| S.D. | 0.58 | 2.6359 | 58.14 |
| Average | 2.77 | 14.0974 | 237.37 |
| CV(%) | 20.88 | 18.6977 | 24.50 |
